# Calibration Method of Orthogonally Splitting Imaging Pose Sensor Based on KDFcmPUM

**DOI:** 10.3390/s19224991

**Published:** 2019-11-16

**Authors:** Na Zhao, Changku Sun, Peng Wang

**Affiliations:** 1 State Key Laboratory of Precision Measuring Technology and Instruments, Tianjin University, Tianjin 300072, China; zhaona@tjau.edu.cn (N.Z.); sunck@tju.edu.cn (C.S.); 2 College of Computer and Information Engineering, Tianjin Agricultural University, Tianjin 300384, China

**Keywords:** KDFcmPUM, orthogonally splitting imaging pose sensor, calibration method

## Abstract

This paper proposes a partition of unity method (PUM) based on KDFCM (KDFcmPUM) that can be implemented to solve the dense matrix problem that occurs when the radial basis function (RBF) interpolation method deals with a large amount of scattered data. This method introduces a kernel fuzzy clustering algorithm to improve clustering accuracy and achieve the partition of unity. The local compact support RBF is used to construct the weight function, and local expression is obtained from the interpolation of the global RBF. Finally, the global expression is constructed by the weight function and local expression. In this paper, the method is applied to the orthogonally splitting imaging pose sensor to establish the mathematical model and the calibration and test experiments are carried out. The calibration and test accuracy both reached ±0.1 mm, and the number of operations was reduced by 4% at least. The experimental results show that KDFcmPUM is effective.

## 1. Introduction

In order to achieve pose measurement with a wide range and high precision, the orthogonally splitting imaging pose sensor uses dual linear CCDs (charge-coupled devices) to simulate a single array CCD. The optical structure is specially designed for dual linear CCDs, so the optical structure and assembly structure are more complicated. As such, the pose sensor has various distortions, such as radial distortion caused by a wide field of view, unidirectional distortion caused by a cylindrical mirror, and nonlinear distortion caused by assembly. Distortion correction must be performed before calibration to achieve high precision measurement. Yang [1] used polynomials to solve distortion parameters for distortion correction. However, distortion correction cannot solve all distortions in the optical system, and the distortion correction process is complicated. The existing calibration method [2,3] cannot satisfy the calibration of the orthogonally splitting imaging pose sensor.

The general imaging model is a mathematical black box model that does not consider distortion. It can be applied to the orthogonally splitting imaging pose sensor. The pose sensor can be calibrated by RBF interpolation [4]. The computational complexity is $O(M^{3})$, and the camera matrix contains (M+3)×6 unknown parameters, where M is the number of control points, N is the number of scattered data, and M = 0.5 N. It is necessary to increase the number of scattered data to improve the calibration accuracy; the number of control points will increase accordingly. Correspondingly, the number of unknown parameters will increase and computational complexity will be increased. Therefore, the calibration method of the orthogonally splitting imaging pose sensor needs to interpolate with large scattered data.

For interpolating with large scattered data, common numerical methods [5,6] are the finite element method (FEM) and meshless method (MM). The main idea of FEM is to divide the region into a finite number of grids and construct a local approximation function with each grid and points in the grid. FEM transforms continuous problems into discrete problems, with strict requirements for the division of the grid that require a large amount of processing time. It is necessary to continuously re-divide the grid to ensure accuracy with a large range of physical changes, so the operation efficiency is low.

MM solves the grid dependency shortcoming of FEM. According to the different construction methods of the approximation function, numerical methods based on MM mainly include radial basis function (RBF) interpolation [7] and moving least-squares (MLS) [8], as well as the reproducing kernel particle method (RKPM) [9] and the partition of unity method (PUM). 

The research shows that the results of RBF interpolation are the most satisfactory of the various scattered data interpolation methods [10]. Data reduction is the main technical difficulty restricting the improvement of RBF interpolation efficiency. The greedy method is a data reduction method proposed by Rendall [11,12] that selects points with the maximum displacements for RBF interpolation. Michler [13], Sheng [14], and Kedward [15] conducted further research on this basis, and their works are mainly applied to mesh deformation technology in the field of computational fluid dynamics. The greedy method requires N pairs of points from different positions on a single moving surface to form a point set, and uses a single set of control points to construct interpolation of the surface deformation. The method is not suitable for the interpolation with a single surface that is not moving. 

The PUM is another data reduction method for large scattered data. The method partitions domains into sub-domains. RBF interpolation is used to display local features in the sub-domains, then local features are restored globally by a weight function. This method is widely used in image segmentation [16,17,18] and three-dimensional surface reconstruction [19,20,21]. Different methods have been proposed to partition the domain and construct the weight function to improve the PUM’s management of large scattered data. In 2001, Beatson [22] proposed an alternating projection method with a multiplicative Schwarz-type method to partition the domain that can improve the efficiency of RBF interpolation with a large dataset, but the process is complicated. Octrees were proposed by Ohtake for domain partitioning [23], using a piecewise polynomial function as local expression and B-spline curves as the weight function in order to solve the problem of local information expression and 3D image reconstruction. However, the weight function was so complicated that it was difficult to implement. OctPUM was first proposed by Macri [24] in 2007. The method is based on quadtrees and octrees, which can be applied to partition two-dimensional or three-dimensional domains. A quartic spline was chosen to construct the leaf nodes of the quadtrees and octrees. The method could permit the analog reconstruction of local information of the three-dimensional image, but the storage structure is complicated and takes up a large amount of memory space (the computational efficiency is also low). In 2010, the parallel algorithm with restricted additive Schwarz method was proposed by Yokota [25] to improve computational efficiency of RBF interpolation. In 2016, the average partition method used to partition the domain was proposed by Li Sen [26], using a weight function constructed according to the Shepard method with a Wendland function [27]. The method was applied to the study of piezoelectric structures. In 2017, a local compactly supported RBF proposed by Wendland was used for the interpolation of a triangular domain by Skala [28]. In 2018, a divide-and-conquer algorithm for domain partition was proposed by Smolik [16] in which the sub-domains can be arbitrary shapes, the local expressions are represented by the local compactly supported RBF proposed by Wendland, and the weight functions are constructed by bilinear interpolation. When the sub-domain does not contain data points or contains all points, it needs to be repartitioned, although the steps are complicated.

The existing methods do not consider the distribution of scattered data when performing domain partition, so sub-domains do not necessarily have enough data to express local features. In some studies, the construction method of the weight function is so complicated that it is difficult to implement. Therefore, this paper proposes a calibration method based on KDFcmPUM using fuzzy clustering for domain partition, global RBF for local expression, and local compactly supported RBF for the weight function. The method can solve the calibration problem of the orthogonally splitting imaging pose sensor when confronted with large scattered data, and improve computational efficiency.

## 2. Materials and Methods

### 2.1. Partition of Unity Method Based on KDFcm (KDFcmPUM)

#### 2.1.1. Fuzzy C-Means Clustering Based on Kernel Distance (KDFcm)

The main idea of the fuzzy c-means clustering algorithm (FCM) is to calculate the distance-weighted sum of squares from each point to the cluster center, and to use the degree of membership to describe the extent to which each point belongs to the class. The algorithm can avoid the error caused by directly dividing a point into a certain class. According to the objective function given by Bezdek [29], the cluster center and the degree of membership matrix can be obtained according to the optimal solution of Equation (1). FCM clustering results can be obtained through iterations until the error requirement is met.

(1) $$J_{m}=\sum_{i=1}^{N}\sum_{j=1}^{k}\mu_{ij}^{m}{\left\|x_{i}-c_{j}\right\|}^{2}$$

where *N* is the number of image points; *k* is the number of clusters, *m* is the weighted index; *x_i_* is the image point; *c_j_* is the cluster center; and $\mu_{ij}$s the degree of membership of the scattered point *i* to the cluster center *j*, which satisfies Equation (2).

(2) $$\sum_{j=1}^{k}\mu_{ij}=1$$

Therefore, the FCM is sensitive to noise and outliers. The clustering results of the FCM are affected by the initial values of the cluster center and the degree of membership matrix, so the global optimal solution cannot be guaranteed. In order to improve the robustness of the FCM, the KDFcm algorithm relaxes the constraint of the degree of membership and remaps the FCM distance function. The algorithm uses the mapping function φ to define the kernel function *K*, allowing Equation (1) to be rewritten as Equation (3).

(3) $$J_{m}=\sum_{i=1}^{N}\sum_{j=1}^{k}\mu_{ij}^{m}\left\|\phi (x_{i})-\phi (c_{j})\right\|$$

(4) K(x,y)=ϕ(x)ϕ(y)

Use Equation (4) to rewrite Equation (3) as Equation (5).

(5) $$J_{m}=\sum_{i=1}^{N}\sum_{j=1}^{k}\mu_{ij}^{m}(K(x_{i},x_{i})+K(c_{j},c_{j})-2K(x_{i},c_{j}))$$

In Equation (5), the Kernel function *K* is a Gaussian Kernel function, as shown in Equation (6), and α is the shape parameter of the Kernel function.

(6) $$K(x,y)=e^{-{\left\|x-y\right\|}^{2}/2\alpha^{2}}$$

According to Equation (6), Equation (5) can be rewritten as Equation (7).

(7) $$J_{m}=\sum_{i=1}^{N}\sum_{j=1}^{k}\mu_{ij}^{m}(2-2K(x_{i},c_{j}))$$

According to the Lagrange multiplier method, the degree of membership is derived from Equation (7), as shown in Equation (8).

(8) $$\mu_{ij}=\frac{{\left(1-K(x_{i},c_{j})\right)}^{\frac{-1}{m-1}}}{\sum_{l=1}^{k}{\left(1-K(x_{i},c_{l})\right)}^{\frac{-1}{m-1}}}$$

The clustering center *c_j_* can be expressed as Equation (9).

(9) $$c_{j}=\frac{\sum_{i=1}^{N}\mu_{ij}K(x_{i},c_{j})x_{i}}{\sum_{i=1}^{N}\mu_{ij}K(x_{i},c_{j})}$$

The following is a description of the KDFcm algorithm.

Step 1: The initial value of the clustering center set $C^{\left(0\right)}=\left\{c_{j}|1\leq j\leq k\right\}$ is obtained by FCM;

Step 2: Parameters are chosen for the selected kernel function *K*, the number of clusters *k*, the weighted index *m*, and the convergence accuracy ε;

Step 3: The degree of membership $\mu_{ij}$ is calculated according to Equation (8) as the initial value of the degree of membership matrix $U^{\left(0\right)}$;

Step 4: The cluster center set $C^{\left(p\right)}$ is calculated according to Equation (9), where *p* is the number of iterations and the cluster center is updated with the latest values; 

Step 5: The degree of membership matrix $U^{\left(p\right)}$ is recalculated based on the updated cluster center;

Step 6: The fourth and fifth steps are performed iteratively until the degree of membership matrix satisfies the requirement of convergence accuracy, or there exists a scattered point that satisfies Equation (2).

According to the algorithm steps, the computational complexity of KDFcm is $O(N^{2})$. 

#### 2.1.2. Partition of Unity

This paper proposes a partition of unity method based on KDFcm that can reflect the distribution characteristics of scattered data. Firstly, cluster the image points by KDFcm. According to the cluster center and the distribution of image points, partition the domain *E* into sub-domain $E_{i}$, which is a circular area and thus satisfies Equation (10).

(10) $$E=\sum_{i=1}^{k}E_{i}$$

Any point on the domain to be interpolated has a weight $\mu_{i}(x)$ corresponding to each sub-domain, and the weight of that point satisfies $\sum_{i=1}^{k}\mu_{i}(x)=1$. 

The local expression $f_{i}(x)$ can be solved by RBF interpolation in the sub-domain, so the global expression can be shown as Equation (11).

(11) $$f(x)=\sum_{i=1}^{k}f_{i}(x)\mu_{i}(x)$$

**Figure 1:**
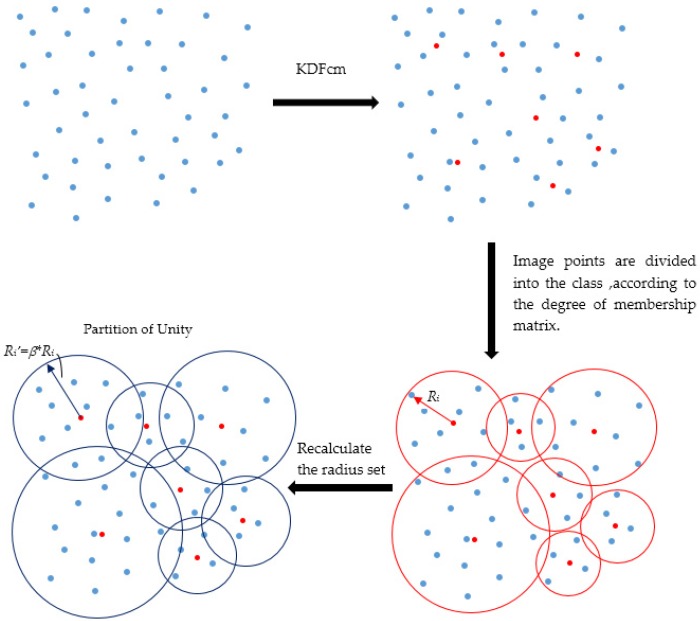
The partition of unity process. In the figure, KDFcm is the abbreviation of fuzzy c-means clustering based on kernel distance.

According to the distribution characteristics of scattered data, KDFcm can obtain only the cluster center and degree of membership, and the image points are not divided into a class. As a result, the range of each sub-domain cannot be obtained. According to the definition of degree of membership, the degree of membership is higher, and the probability that the image point belongs to a class is greater. Therefore, the image points are divided into the class with the largest degree of membership. The schematic of the process is shown in Figure 1. The steps of the partition of unity are as follows.

Step 1: Determine the number of clusters. According to the number of calibration points, the number of clusters *k* is selected. A big value of *k* will cause few points in the class to represent the local features of the sub-domain. A small value of *k* will result in a lot of points in the class and increase the computational complexity.

Step 2: Establish the KDFcm cluster. The cluster center is set and the degree of membership matrix is determined. (The cluster method is mentioned in Section 2.1.1).

Step 3: Image points are divided into class, according to the degree of membership matrix. The maximum distance from the image point in the class to the cluster center is the initial radius of the class. The radius set is determined by $\left\{R_{i}|i=1,\ldots ,k\right\}$.

Step 4: The partition of unity is carried out. According to the radius set, the sub-domains are generated. If the sum of the sub-domains can cover the entire domain to be interpolated, the partition of unity ends; if not, Step 5 needs to be performed to recalculate the radius set.

Step 5: The radius set is recalculated, if necessary. Since the points near the class can be approximated as having similar characteristics to the class, the initial radius can be multiplied by the radius expansion coefficient β to cover the entire domain. However, there is no standard for the selection of the radius expansion factor, which needs to be adjusted according to the experimental result. Step 4 is repeated.

### 2.2. Mathematical Model

**Figure 2:**
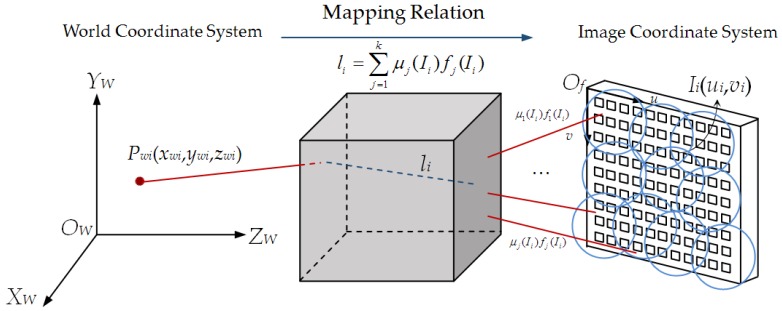
The diagram of mathematical model of the orthogonally splitting imaging pose sensor based on KDFcmPUM. In the figure, KDFcmPUM is the abbreviation of the partition of unity method based on KDFcm).

The mathematical model of the orthogonally splitting imaging pose sensor is established based on KDFcmPUM, as shown in Figure 2. The blue circles in the diagram show the circular sub-domains obtained by KDFcmPUM. The mapping relation $l_{i}$ between $P_{wi}$ in the world coordinate system and $I_{i}$ in the image coordinate system can be described with the local expression $f_{j}(x)$ and the weight function $\mu_{j}(x)$. 

The entire KDFcmPUM process can be split into four stages, listed below.

Stage 1: Partition of unity based on KDFcm.
Stage 2: Construction of the weight function.
Stage 3: Solvingthe local expression with interpolation.
Stage 4: Construction of the global expression.

#### 2.2.1. Construction of Weight Function

After the partition of unity, local features are merged into a global feature by the weight function. In this paper, a compactly supported non-negative continuous function is used to construct the weight function whose radius of influence is the radius set of the circular sub-domains generated by the KDFcmPUM introduced in Section 2.1.2. The weight function $\mu_{i}(x)$ is constructed by the Shepard method [30], as shown in Equation (12).

(12) $$\mu_{i}(x)=\frac{\varphi_{i}(x)}{\sum_{j=1}^{k}\varphi_{j}(x)}$$

where $\varphi_{i}(x)$ is the compactly supported non-negative continuous function, and the derivation of its expression is as follows. 

In order to ensure the continuity of the function, the local compactly supported RBF proposed by Wu [31] is selected as the compactly supported non-negative continuous function to construct the weight function, as shown in Equation (13).

(13) $$\varphi (r)=\{\begin{array}{cc} {\left(1-r\right)}_{+}^{6}(5r^{5}+30r^{4}+72r^{3}+82r^{2}+36r+6) & 0\leq r\leq 1\\ 0 & r>1 \end{array}$$

where *r* can be expressed as Equation (14).

(14) $$r=\frac{\left\|x-c_{i}\right\|}{R_{i}}$$

In Equation (14), ***c****_i_* is the cluster center of the sub-domain $E_{i}$ and *R_i_* is the radius of the circular sub-domain.

Substituting Equation (14) into Equation (12), the weight function is obtained as shown in Equation (15).

(15) $$\mu_{i}(x)=\frac{\varphi (\frac{\left\|x-c_{i}\right\|}{R_{i}})}{\sum_{j=1}^{k}\varphi (\frac{\left\|x-c_{j}\right\|}{R_{j}})}$$

#### 2.2.2. Local Expression

The local expression $f_{j}(x)$ can be expressed by six homogeneous coordinates in the five-dimensional projective space by introducing the Plücker coordinates to represent the mapping relation between the world coordinate system and the image coordinate system. The local expression can be expressed by RBF as Equation (16).

(16) $$f_{j}(x)={\left(s_{1}^{\left(j\right)}(x),s_{2}^{\left(j\right)}(x),s_{3}^{\left(j\right)}(x),s_{4}^{\left(j\right)}(x),s_{5}^{\left(j\right)}(x),s_{6}^{\left(j\right)}(x)\right)}^{T}$$

where $s_{i}(x)$ is the RBF interpolation expression, as shown in Equation (17).

(17) $$s(x)=a_{0}+a_{x}^{T}x+\sum_{I=1}^{M}w_{i}\phi (\left\|x-control_{i}\right\|)$$

where $a_{0}$, $a_{x}$, and $w_{i}$ are unknown parameters, and $control_{i}$ is the control point. Substituting Equation (17) into Equation (16) and converting it into the form of a matrix, Equation (18) can be obtained.

(18) $$f_{j}(x)={\left(\left(\begin{array}{cc} \phi (x) & p(x) \end{array}\right)H_{cam}^{\left(j\right)}\right)}^{T}$$

where ϕ(x) is the RBF,p(x) is the homogeneous coordinates of the 2D image, and $H_{cam}^{\left(j\right)}$ is the local camera matrix of the sub-domain *j*.

#### 2.2.3. Global Expression

Combining the local expression and weight function together, the global expression can be obtained. Substitute Equation (18) into Equation (11) to obtain the global expression, as shown in Equation (19).

(19) $$f(x)=\sum_{j=1}^{k}\mu_{j}(x){\left(\left(\begin{array}{cc} \phi (x) & p(x) \end{array}\right)H_{cam}^{\left(j\right)}\right)}^{T}$$

For an improved interpolation effect, the global RBF (multi-quadric function and Gaussian function) with shape parameters is selected as the interpolation function, as shown in Table 1. In the basis function, *α* is the shape parameter. However, there is no method to calculate the value of the shape parameter, so it is necessary to obtain a reasonable value of the shape parameter according to the experimental result.

In summary, for a given RBF and its shape parameter, the image points set $\left\{x_{i}|i=1,\ldots ,N\right\}$, control points set $\left\{c_{i}|i=1,\ldots ,M\right\}$, camera matrix $H_{cam}^{\left(j\right)}$, and local expression $f_{j}(x)$ of the sub-domain can be estimated. Based on the cluster center set and the radius of influence set, the global expression f(x) can be estimated. Only the camera matrix $H_{cam}^{\left(j\right)}$ of the sub-domain is unknown and needs to be calibrated.

**Figure 3:**
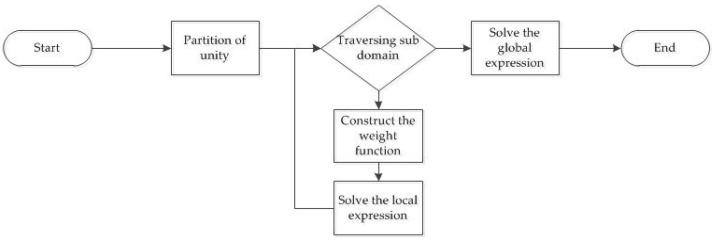
The KDFcmPUM process.

The entire KDFcmPUM process is shown in Figure 3. According to the computational complexity of the four stages, the entire computational complexity of the KDFcmPUM process is $O(N^{2})+O(N^{2})+\sum_{i=1}^{k}O(M_{i}^{3})+O(N)$, where $\sum_{i=1}^{k}M_{i}=M=N/2$. As such, the computational complexity of KDFcmPUM is smaller than $O(M^{3})$, and the number of operations is reduced by 4% at least for N = 1008. KDFcmPUM is more efficient than the method based only on RBF interpolation [4].

## 3. Results

### 3.1. Experimental Apparatus

**Figure 4:**
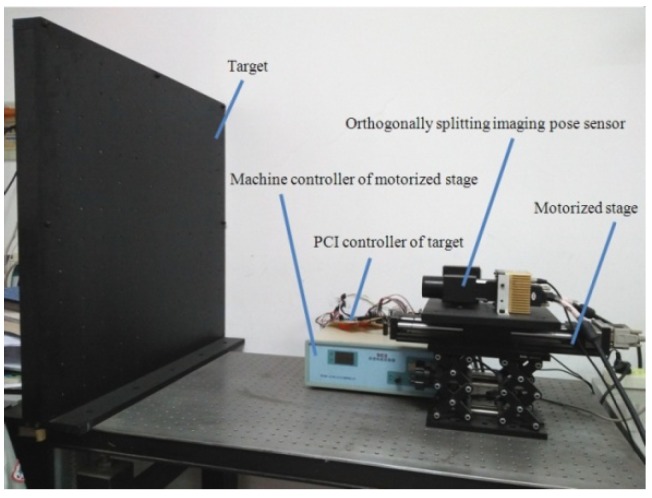
Experimental apparatus.

The experimental apparatus consisted of the target, the PCI controller of the target, the motorized stage, the mechanized controller of the motorized stage, and the orthogonally splitting imaging pose sensor. The target was a plate with 12 × 12LEDs representing the horizontal and vertical distance between the point, respectively, with the point being 60 mm. The PCI controller of the target controlled the LEDs and cause them to light up at different times. The motorized stage was TSA-200, of which the stroke was 200 mm, the resolution under the eight sub-divisions was 2.5 μm, and the repeat positioning accuracy was greater than 5 μm. The orthogonally splitting imaging pose sensor was fixed on the motorized stage and could be moved by the mechanized controller. The experimental apparatus is shown in Figure 4.

### 3.2. Experiment Data

The data set consisted of image coordinates and world coordinates of *n* points, recorded as $\left\{x_{i}\to w_{i}|i=1,\ldots ,n\right\}$. The acquisition method of the data set was as follows.

In the initial position, LEDs on the target lit up at different times, and the orthogonally splitting imaging pose sensor sequentially acquired the image coordinates of the points, with pixels as data unit. The relative world coordinates of the points on the target were simultaneously recorded in this position, with mm as the unit of measurement. Translational motion of the orthogonally splitting imaging pose sensor changed the relative position of the target. The relative world coordinates and image coordinates of the points were acquired according to the method in the initial position. The above steps were repeated several times to acquire the data set.

#### 3.2.1. Calibration Data Set

**Figure 5:**
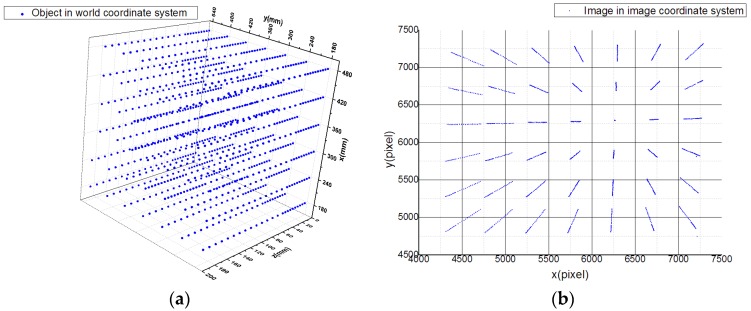
Calibration data set: (**a**) world coordinates and (**b**) image coordinates.

According to the above acquisition method, the calibration data set consisted of 1008 points. The image coordinates and world coordinates of the data set are shown in Figure 5. 

#### 3.2.2. Test Data Set

**Figure 6:**
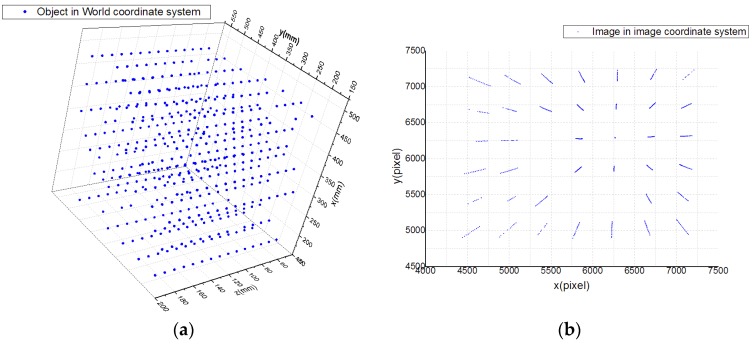
Test data set: (**a**) world coordinates and (**b**) image coordinates.

To test the accuracy of the calibration results, a test data set was used. According to the above acquisition method, the test data set consisted of 448 points, and there is no intersection between the calibration data set and the test data set. The image coordinates and world coordinates of the data set are shown in Figure 6. 

### 3.3. Calibration and Test

#### 3.3.1. KDFcmPUM

**Figure 7:**
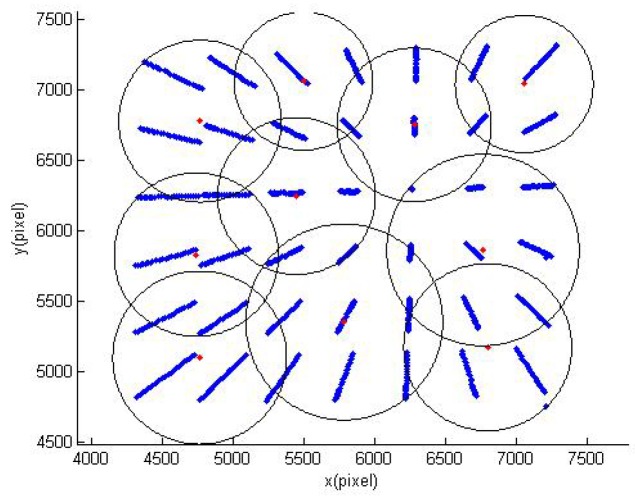
The initial partition of unity.

**Figure 8:**
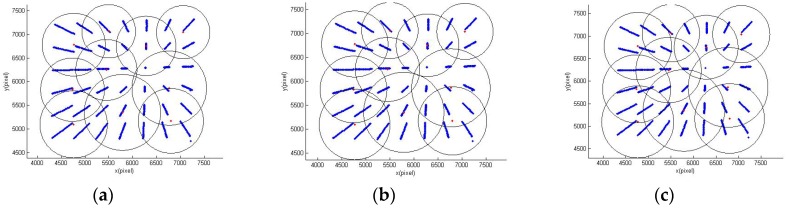
Partition of unity with different radius expansion coefficients: (**a**) *β* = 1.15, (**b**) *β* = 1.2, and (**c**) *β* = 1.3.

According to the KDFcmPUM introduced in Section 2.1, KDFcm was performed on the image coordinates of the points in the calibration data set, and the cluster center set and circular sub-domain’s radius set were obtained to perform the initial partition of unity, as shown in Figure 7. The initial partition of unity could not cover the entire domain to be interpolated, so the radius *r* needed to be recalculated with the radius expansion coefficient *β*(1<β<2 in order to update the partition of unity with different radius expansion coefficients, as shown in Figure 8a–c.

#### 3.3.2. Parameter Experiment

As described in Section 2.2.2, the shape parameters of the RBF used in the local expression need to be selected according to the experimental results. Therefore, different values were used to calibrate and test according to the partition of unity, as seen in Figure 8 and Table 1. The error evaluation includes two parts: (1) the evaluation of the calibration error and test error according to the distance between the object point in the world coordinate system and the fitting Plücker line; and (2) the evaluation of the fitting error of the world coordinates according to the distance between the ideal world coordinates and the fitting world coordinates. The errors of different radius expansion coefficients on calibration accuracy are evaluated, and reasonable values of the shape parameters are selected for different RBFs.

RBF interpolation with multi-quadratic (MQ) function

**Figure 9:**
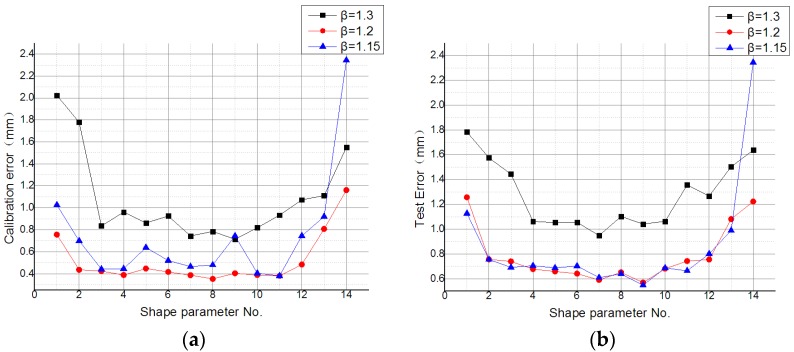
Calibration error and test error of the MQ function with different values of the shape parameter in different partition of unity for the (**a**) calibration data set and (**b**) test data set.

This paper evaluates the errors of the MQ function for different values of the shape parameter *α* in different partitions of unity. Test error trends for different shape parameters can be seen in Figure 9. In the figure, the x-axis is the shape parameter number, and the y-axis is the error value. As shown in Figure 9a, the calibration errors of numbers 2 and 3 were less than 1 mm. As such, the range of the shape parameter’s value was [0.0001, 1]. These values of the shape parameter will be used in subsequent experiments.

**Figure 10:**
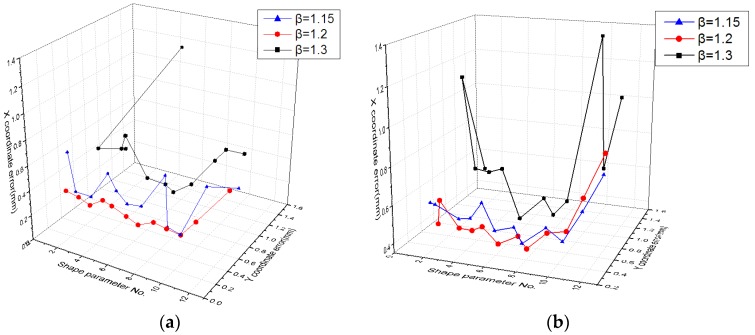
Fitting error of the MQ function with different values of the shape parameter in different partitions of unity for (**a**) the calibration data set and the (**b**) test data set.

The effect of different values of the radius expansion coefficient *β* on the error evaluation was studied, and it can be clearly seen from Figure 9 and Figure 10 that error evaluation of *β* = 1.3 was higher than the other two cases. This means that the overlap of each sub-domain was not too large. However, although *β* = 1.15 can satisfy the minimum sub-domain overlap, the error evaluation is not at its lowest, indicating that reasonable sub-domain overlap is necessary. Therefore, a partition of unity with *β* = 1.2 can achieve a lower error evaluation.

**Figure 11:**
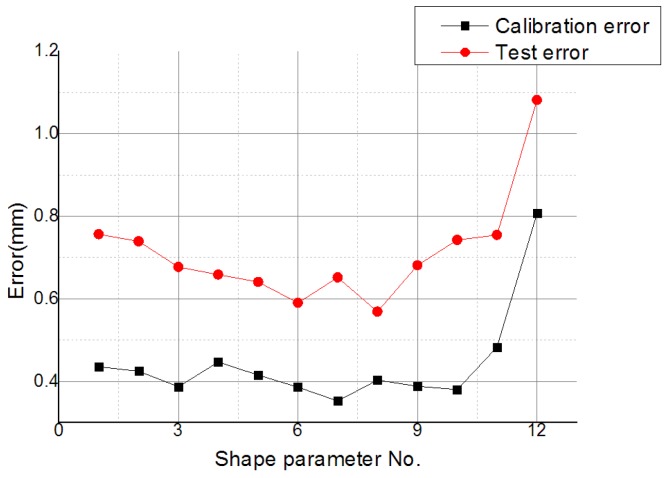
Calibration error and test error with different values of the shape parameter.

The effect of different values of the shape parameter on the error evaluation in a partition of unity with *β* = 1.2 was studied, and the calibration errors of 12 shape parameter values were lower than 1 mm, with test errors varying along with calibration errors, as shown in Figure 11. The reasonable value of shape parameter is the value withthe lowest calibration error and the test error closest to the calibration error. As such, number 8 (*α* = 0.095) was selected as the reasonable value. The fitting error of number 8 was lower than the others, as shown in Table 2. 

RBF interpolation with Gaussian function

Using the same MQ function method to select the value of the shape parameter, the range of the shape parameter’s value was determined to be [0.01, 0.1], and nine shape parameter values were selected for subsequent experiments. 

**Figure 12:**
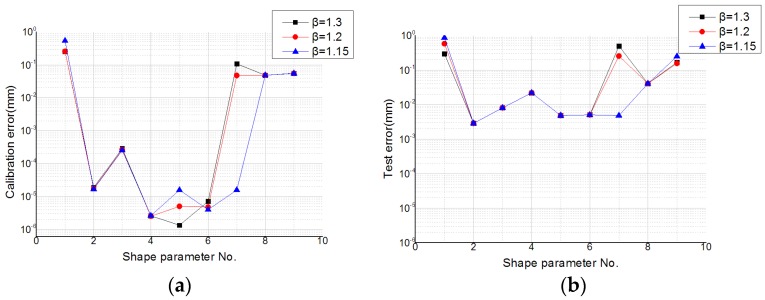
Calibration error and test error of the Gaussian function with different values of the shape parameter in different partitions of unity for (**a**) the calibration data set and (**b**) the test data set. The y-axis uses a logarithmic scale.

**Figure 13:**
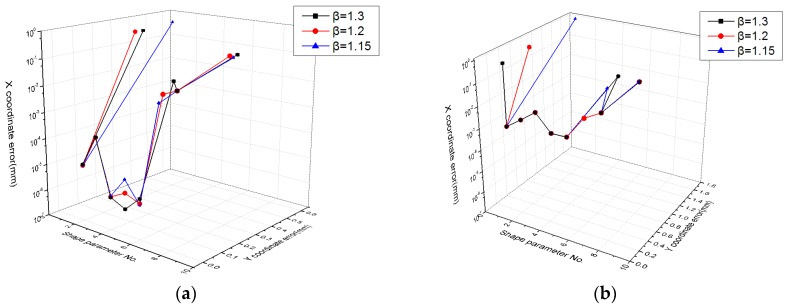
Fitting error of the Gaussian function with different values of the shape parameter in different partition of unity for (**a**) the calibration data set and (**b**) the test data set. The y-axis uses a logarithmic scale.

The effect of different values of the radius expansion coefficient *β* on the error evaluation was studied. It can be clearly seen from Figure 12 that interpolation with the Gaussian function had a less calibration error and test error. The calibration errors of numbers 2–6 were close, and the test errors were nearly equal. Figure 12 and Figure 13 show that the error evaluation of *β* = 1.3 was higher than the other two cases. Similar results to the MQ function can be seen in Figure 9 and Figure 10. The partition of unity with *β* = 1.2 was able to achieve a lower error evaluation.

**Figure 14:**
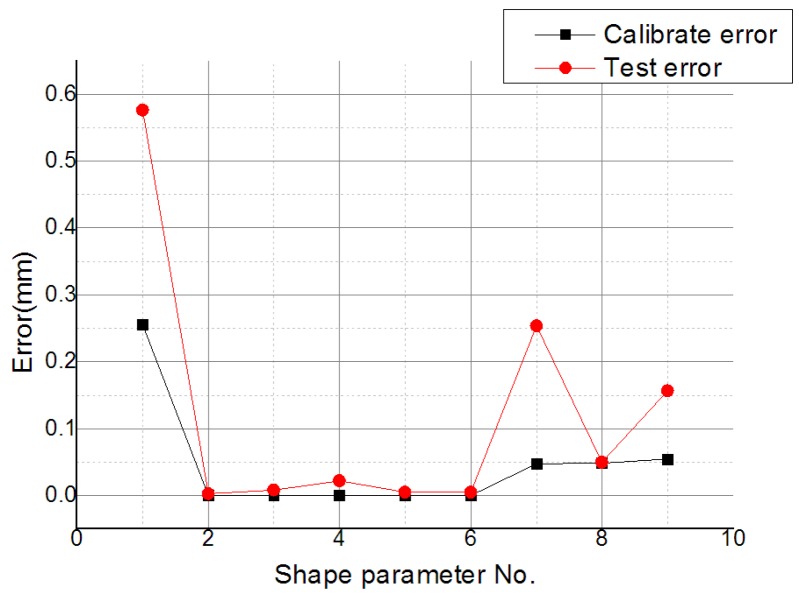
Calibration errors and test errors with different values of the shape parameter.

The effect of different values of the shape parameter on the error evaluation in the partition of unity with *β* = 1.2 was studied. The calibration errors of numbers 2–9 of the shape parameter’s values were lower than 0.1 mm, and test errors varied with the calibration errors, as shown in Figure 14. For the selection of the MQ function, number 6 (*α* =0.055) was selected as the reasonable value. The fitting error of number 6 was lower than the others, as shown in Table 3.

Comparison with different RBFs

Calibration errors and test errors are shown with different RBFs in Table 4. Calibration with the Gaussian function was able to obtain less calibration and test errors than calibration with the MQ function. Calibration accuracy satisfied the requirements of the orthogonally splitting imaging pose measurement system (0.1 mm). 

### 3.4. Comparative Experiment

As the amount of data in the calibration data set increases, the accuracy of the calibration method increases. For this reason, calibration was performed with different calibration data sets using the same test data set. The experimental results are shown in Table 5. Due to the reduced computational complexity, KDFcmPUM operated at a higher efficiency than RBF. The increase of data was able to effectively improve calibration accuracy of the orthogonally splitting imaging pose sensor, and KDFcmPUM was able to satisfy the calibration requirements of large scattered data while improving calibration efficiency.

## 4. Discussion

This paper proposed using KDFcmPUM to solve the calibration problem of the orthogonally splitting imaging pose sensor with large scattered data. The method uses the KDFcm for the partition of unity in order to properly reflect the distribution of scattered data. A local RBF was introduced to construct the weight function, and global RBF interpolation was used to obtain the local expression. The weight function and local expression were then used to construct a global expression. Correspondingly, the mathematical model of the orthogonally splitting imaging pose measurement system was established. The radius expansion coefficient was selected through experimentation, and the orthogonally splitting imaging pose sensor was calibrated and tested based on the partition of unity. A reasonable value for the shape parameter was selected through experiments to meet the accuracy of the measurement system. The experimental results demonstrate the effectiveness of KDFcmPUM. The method can process large scattered data and improve the calibration accuracy and efficiency of the orthogonally splitting imaging pose sensor. In the future, the influence of the number of clusters on the calibration can be further studied, and the application of the method to different fields can be studied.

## Figures and Tables

**Table 1:** Global RBF (Radial Basis Function).

Global RBF	Basis Function
Multi-quadric (MQ) function	(r2+α2)12
Gaussian function	$e^{-\alpha^{2}r^{2}}$

**Table 2:** Fitting error with different values of the shape parameter.

No.	Calibration Data Set		Test Data Set	
	$x_{w}\;(\mathrm{mm})$	$y_{w}\;(\mathrm{mm})$	$x_{w}\;(\mathrm{mm})$	$y_{w}\;(\mathrm{mm})$
1	0.32	0.30	0.65	0.39
2	0.33	0.27	0.46	0.58
3	0.31	0.24	0.54	0.42
4	0.32	0.31	0.51	0.43
5	0.27	0.32	0.43	0.48
6	0.29	0.26	0.45	0.40
7	0.27	0.23	0.53	0.42
8	0.30	0.27	0.41	0.41
9	0.29	0.25	0.50	0.47
10	0.30	0.23	0.58	0.47
11	0.32	0.37	0.59	0.65
12	0.64	0.50	0.71	0.86

**Table 3:** Fitting errors with different values of the shape parameter.

No.	Calibration Data Set		Test Data Set	
	$x_{w}\;(\mathrm{mm})$	$y_{w}\;(\mathrm{mm})$	$x_{w}\;(\mathrm{mm})$	$y_{w}\;(\mathrm{mm})$
1	3.60E-01	3.46E-01	6.98E-01	6.81E-01
2	1.04E-05	1.39E-05	6.16E-05	2.87E-03
3	1.40E-04	2.23E-04	2.68E-04	8.29E-03
4	1.71E-06	1.85E-06	5.62E-04	2.56E-02
5	3.25E-06	3.81E-06	5.23E-05	4.85E-03
6	4.24E-06	2.28E-06	1.46E-04	5.10E-03
7	4.29E-02	2.00E-02	2.99E-02	4.19E-02
8	3.29E-02	3.54E-02	3.97E-02	9.81E-02
9	2.20E-01	3.27E-01	4.51E-01	6.17E-01

**Table 4:** Comparison with different RBFs.

RBF	Calibration Data Set (mm)	Test Data Set (mm)
MQ function	0.74	0.55
Gaussian function	4.81 × 10^−6^	0.005

**Table 5:** Comparison with different calibration methods.

Calibration Method	Test Data Set (mm)	Number of Points in Calibration Data Set	Operation Time (s)
RBF	0.022	700	51.4
KDFcmPUM	0.005	1008	7.7
